# Novel Thermal Diffusion Temperature Engineering Leading to High Thermoelectric Performance in Bi_2_Te_3_‐Based Flexible Thin‐Films

**DOI:** 10.1002/advs.202103547

**Published:** 2021-12-22

**Authors:** Dong‐Wei Ao, Wei‐Di Liu, Yue‐Xing Chen, Meng Wei, Bushra Jabar, Fu Li, Xiao‐Lei Shi, Zhuang‐Hao Zheng, Guang‐Xing Liang, Xiang‐Hua Zhang, Ping Fan, Zhi‐Gang Chen

**Affiliations:** ^1^ Shenzhen Key Laboratory of Advanced Thin Films and Applications Key Laboratory of Optoelectronic Devices and Systems of Ministry of Education and Guangdong Province College of Physics and Optoelectronic Engineering Shenzhen University Shenzhen 518060 P. R. China; ^2^ Centre for Future Materials University of Southern Queensland Springfield Central Brisbane QLD 4300 Australia; ^3^ School of Mechanical and Mining Engineering The University of Queensland St Lucia QLD 4072 Australia; ^4^ Univ Rennes CNRS ISCR (Institut des Sciences Chimiques de Rennes) UMR6226 Rennes F‐35000 France

**Keywords:** Bi_2_Te_3_, thermal diffusion method, thermoelectrics, flexible thin films

## Abstract

Flexible Bi_2_Te_3_‐based thermoelectric devices can function as power generators for powering wearable electronics or chip‐sensors for internet‐of‐things. However, the unsatisfied performance of n‐type Bi_2_Te_3_ flexible thin films significantly limits their wide application. In this study, a novel thermal diffusion method is employed to fabricate n‐type Te‐embedded Bi_2_Te_3_ flexible thin films on flexible polyimide substrates, where Te embeddings can be achieved by tuning the thermal diffusion temperature and correspondingly result in an energy filtering effect at the Bi_2_Te_3_/Te interfaces. The energy filtering effect can lead to a high Seebeck coefficient ≈160 µV K^−1^ as well as high carrier mobility of ≈200 cm^2^ V^−1^ s^−1^ at room‐temperature. Consequently, an ultrahigh room‐temperature power factor of 14.65 µW cm^−1^ K^−2^ can be observed in the Te‐embedded Bi_2_Te_3_ flexible thin films prepared at the diffusion temperature of 623 K. A thermoelectric sensor is also assembled through integrating the n‐type Bi_2_Te_3_ flexible thin films with p‐type Sb_2_Te_3_ counterparts, which can fast reflect finger‐touch status and demonstrate the applicability of as‐prepared Te‐embedded Bi_2_Te_3_ flexible thin films. This study indicates that the thermal diffusion method is an effective way to fabricate high‐performance and applicable flexible Te‐embedded Bi_2_Te_3_‐based thin films.

## Graphical abstract

In this study, flexible *n*‐type Bi_2_Te_3_‐based thin‐films are successfully prepared through facile thermal diffusion method and further induce Te/Bi_2_Te_3_ heterojunctions and energy filtering effect at the Te/Bi_2_Te_3_ interfaces to optimize the thermoelectric performance through tuning the diffusion temperature.

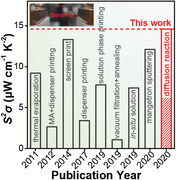

## 1 Introduction

Thermoelectrics, as an emission‐free power generation technology,^[^ ^1^, ^2^, ^3^ ^]^ can provide eco‐friendly power supply for the portable and wearable electronic devices, such as chip‐sensors,^[^ ^4^ ^]^ electrocardiographic systems,^[^ ^5^ ^]^ and implantable electronic devices.^[^ ^6^ ^]^ The conversion efficiency of a thermoelectric device is determined by the performance of composing thermoelectric materials, which is evaluated by dimensionless figure‐of‐merit *ZT* (*ZT* = *S* ^2^ *σT*/*κ*, where *S*, *σ*, *S* ^2^ *σ*, *T*, and *κ* are the Seebeck coefficient, electrical conductivity, power factor, absolute temperature, and thermal conductivity, respectively).^[^ ^7^, ^8^ ^]^ Regardless of high‐performance thermoelectric materials, such as SnSe,^[^ ^9^ ^]^ Cu_2_Se,^[^ ^10^ ^]^ CoSb_3_,^[^ ^11^ ^]^ GeTe,^[^ ^12^ ^]^ and Mg_3_Sb_2_,^[^ ^13^ ^]^ traditional Bi_2_Te_3_‐based thermoelectric materials are still widely studied because of their superior performance at room‐temperature.^[^ ^14^, ^15^, ^16^ ^]^

With potentially high flexibility, thermoelectric thin films are more suitable for wearable electronic design. Particularly, n‐type Bi_2_Te_3_‐based and p‐type Bi_0.5_Sb_1.6_Te_3_‐based thermoelectric materials have attracted extensive attentions.^[^ ^15^, ^16^, ^17^, ^18^, ^19^, ^20^ ^]^ Various material engineering strategies, such as defect‐engineering,^[^ ^21^, ^22^ ^]^ texturing engineering,^[^ ^23^ ^]^ nanoengineering,^[^ ^24^, ^25^, ^26^ ^]^ have been used to boost thermoelectric performance of Bi_2_Te_3_‐based flexible thin films. Till now, significant enhancements have been observed in p‐type Bi_0.5_Sb_1.5_Te_3_‐based thin films. Shang et al.^[^ ^27^ ^]^ employed a magnetron sputtering method to fabricate the p‐type Ag‐doped Bi_0.5_Sb_1.5_Te_3_ films with an optimal *S* ^2^ *σ* of ≈14.0 µW cm^−1^ K^−2^ at 420 K. Varghese et al.^[^ ^28^ ^]^ used a scalable screen‐printing method to prepare p‐type BiSbTe flexible film with a *S* ^2^ *σ* of ≈30 µW cm^−1^ K^−2^ at room temperature. However, high‐performance n‐type Bi_2_Te_3_‐based thermoelectric thin films need further development due to the low *S* ^2^ *σ* (˂14 µW cm^−1^ K^−2 [^ ^28^, ^29^ ^]^) comparing with its p‐type counterparts (the *S* ^2^ *σ* of p‐type Bi_0.5_Sb_1.5_Te_3_‐based thermoelectric thin films can approach as high as ≈20 µW cm^−1^ K^−2 [^ ^26^ ^]^).

To date, numerous methods have been adopted to prepare high‐performance n‐type Bi_2_Te_3_‐based flexible thin films. Bi_2_Te_3_ nanowire‐based flexible thin films prepared by solution phase printing can approach the *S* ^2^ *σ* of ≈1.10 µW cm^−1^ K^−2^ at 400 K.^[^ ^30^ ^]^ Hybridizing Bi_2_Te_3_ with graphene oxide and form flexible thin films by vacuum filtration and annealing also approach the *S* ^2^ *σ* of ≈1.08 µW cm^−1^ K^−2^ at ≈297 K.^[^ ^31^ ^]^ Se‐doping in Bi_2_Te_3_ achieved by mechanical alloying in combination with dispenser printing to form flexible thin films can achieve the *S* ^2^ *σ* to ≈2.65 µW cm^−1^ K^−2^ at ≈297 K.^[^ ^19^ ^]^ Through hybridizing Bi_2_Te_3_ with polyimide via dispenser printing, Jung et al.^[^ ^32^ ^]^ successfully prepared a Bi_2_Te_3_ flexible thin film with the high *S* ^2^ *σ* of ≈3.43 µW cm^−1^ K^−2^ at ≈297 K. Hybridizing carbon nanotubes with Bi_2_Te_3_ and form flexible thin films by in situ solution method successfully enhanced the *S* ^2^ *σ* to ≈7.4 µW cm^−1^ K^−2^ at ≈297 K.^[^ ^33^ ^]^ Additionally, a glass fabric‐based Bi_2_Te_3_ flexible film prepared by screen printing has approached the *S* ^2^ *σ* of ≈13.32 µW cm^−1^ K^−2^ at ≈297 K.^[^ ^34^ ^]^ Most of these methods are suffering from poor crystal growth, which has limited the carrier transport properties and correspondingly limited the thermoelectric performance of as‐prepared Bi_2_Te_3_‐based flexible thin films.

**Figure 1:**
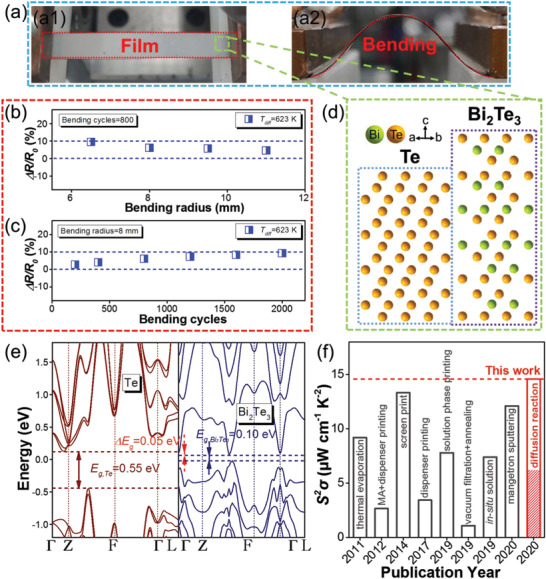
a) Optical images of as‐prepared Bi_2_Te_3_ flexible thin film under (a1) flat and (a2) bending statuses, respectively. b) The Δ*R/R*_0_ of the Bi_2_Te_3_ flexible thin film prepared under the diffusion temperature of 623 K as a function of bending radius at the bending cycles of 800 times. c) The Δ*R*/*R*_0_ of the Bi_2_Te_3_ flexible thin film prepared under the diffusion temperature of 623 K as a function bending cycles at the bending radius of 8 mm. d) Schematic structure of Bi_2_Te_3_ and Te. e) DFT calculated electronic band structures of Te and Bi_2_Te_3_, respectively. f) Comparison of the maximum room‐temperature *S^2^σ* between our Bi_2_Te_3_‐based flexible thin films and other state‐of‐art n‐type Bi_2_Te_3_‐based flexible thin films prepared by different methods, including thermal evaporation (2011),^[^ ^35^ ^]^ mechanically alloyed (MA) and dispenser printing (2012),^[^ ^19^ ^]^ screen print (2014),^[^ ^34^ ^]^ disperser printing (2017),^[^ ^32^ ^]^ solution phase printing (2019),^[^ ^30^ ^]^ vacuum filtration and annealing (2019),^[^ ^31^ ^]^ in situ solution (2019),^[^ ^33^ ^]^ magnetron sputtering (2020),^[^ ^36^ ^]^ and this work.

In this study, to render sufficient energy for Bi_2_Te_3_ crystal growth, we use an advanced thermal diffusion method to fabricate n‐type Bi_2_Te_3_ flexible thin films on flexible polyimide (PI) substrates (**Figure** **1**a). Our prepared Bi_2_Te_3_ flexible thin films (use the film prepared under the diffusion temperature of 623 K as an example) also possess high stability after bending test under various bending radius and bending cycles as evidenced by the minor change of resistance (Δ*R/R*_0_) presented in Figure 1b,c, respectively. Through tuning the diffusion temperature (*T*_diff_), Te‐embedding can form and lead to Bi_2_Te_3_/Te heterostructures (Figure 1d). It can induce energy filtering effect at the Bi_2_Te_3_/Te interfaces due to an energy offset (Δ*E*_g_) between the Te and Bi_2_Te_3_ conduction band minimums as shown in Figure 1e, which is calculated by density functional theory (DFT), and in turn lead to a high Seebeck coefficient ≈160 µV K^−1^ as well as high carrier mobility of ≈200 cm^2^ V^−1^ s^−1^. Correspondingly a high maximum *S^2^σ* of 14.65 µW cm^−1^ K^−2^ can be achieved in the Te‐embedded Bi_2_Te_3_ flexible thin film prepared under the diffusion temperature of 623 K, which is comparable with other Bi_2_Te_3_‐based flexible thin films (Figure 1f). Applicability of as‐prepared n‐type Bi_2_Te_3_ flexible thin films are further demonstrated by an assembled thermoelectric sensor, which can fast reflect the finger touch status.

## 2 Results and Discussion

**Figure 2:**
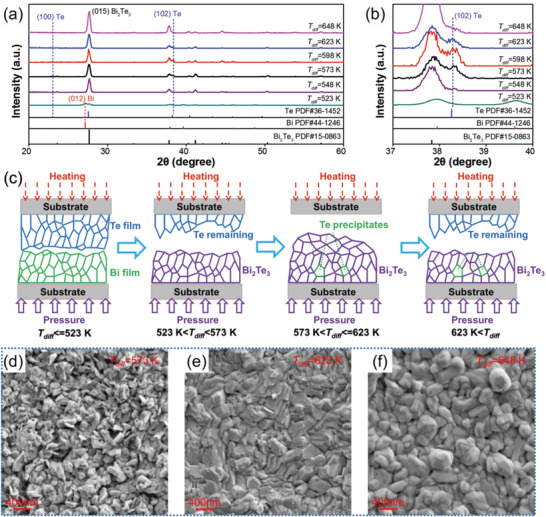
a) XRD patterns of Bi_2_Te_3_‐based flexible thin films prepared under different diffusion temperature and b) the enlarged (102) peak of Te. c) Schematic illustration of Bi_2_Te_3_ flexible thin film preparation through thermal diffusion process. SEM images of the Bi_2_Te_3_‐based flexible thin films prepared at the thermal diffusion temperature of d) 573, e) 623, and f) 648 K, respectively.

To understand the crystal structure of the as‐deposited Bi_2_Te_3_‐based flexible thin films, we first performed XRD analyses and their XRD patterns are shown in **Figure** **2**a. As can be seen, when the thermal diffusion temperature is 523 K, the Bi‐Te reaction is insufficient and large amount of Bi (JCPDS 44‐1246) can be observed as well as the formed Bi_2_Te_3_ (JCPDS 15‐0863). With increasing the thermal diffusion temperature to 548 K, the as‐prepared thin film is mainly composed of Bi_2_Te_3_ phase. Further increasing the temperature to above 573 K, Te precipitates can be observed. When the temperature is above 648 K, almost pure phase Bi_2_Te_3_ can be observed. To clarify the crystal structure of Bi_2_Te_3_‐based thin film prepared by our thermal diffusion method, the enlarged (102) peak was plotted in Figure 2b, where the appearance of Te at the deposition temperature of 573–623 K can be clearly observed. Unobvious Te is observed in the XRD pattern, indicating that there is fewer Te phase above 648 K. Figure 2c schematically shows the thermal diffusion reaction process under different diffusion temperatures. With high saturation vapor pressure and diffusion energy,^[^ ^37^, ^38^ ^]^ Te atoms can easily sublimate and diffuse into the Bi precursor film and form Bi_2_Te_3_ under a high diffusion temperature and high gas pressure. At 523 K, poor Te diffusion leads to poor formation of Bi_2_Te_3_. With increasing the diffusion temperature to 548 K, Te sublimates and reacts with Bi to form Bi_2_Te_3_ more sufficiently. With further increasing the diffusion temperature to 573 K, Te fully sublimates with insufficient Bi_2_Te_3_ formation due to Te over‐sublimation. When the diffusion temperature is as high as 648 K, the Bi precursor film and sublimated Te can fully react to form Bi_2_Te_3_. To render a more detailed semiquantitative analysis of the Te content, the chemical compositions of the Bi_2_Te_3_ thin films prepared at different diffusion temperatures are summarized in Table S1 (Supporting Information). As can be seen, due to insufficient Te diffusion at 523 K, the film is in lack of Te. With the diffusion temperature increased to over 548 K, the Te content increased. Optical images of the Te precursor films after thermal diffusion reaction are shown in Figure S1 (Supporting Information) which demonstrates Te remains under the diffusion temperature of 548 K and fully sublimates at 573 K. Figure 2d–f shows the scanning electron microscope (SEM) images of as‐prepared Bi_2_Te_3_ flexible thin films which are prepared at the diffusion temperature of 573, 623, and 648 K, respectively. The average grain sizes of the Bi_2_Te_3_‐based films prepared at 573, 623, and 648 K are ≈210, ≈285, and ≈310 nm, respectively (detailed size distribution was shown in Figure S2a–c, Supporting Information). The Bi_2_Te_3_‐based flexible thin films are growing denser with increasing diffusion temperature. The increased grain size and density with increasing diffusion temperature should be attributed to faster grain growth rate and element diffusion rate at higher temperature.

**Figure 3:**
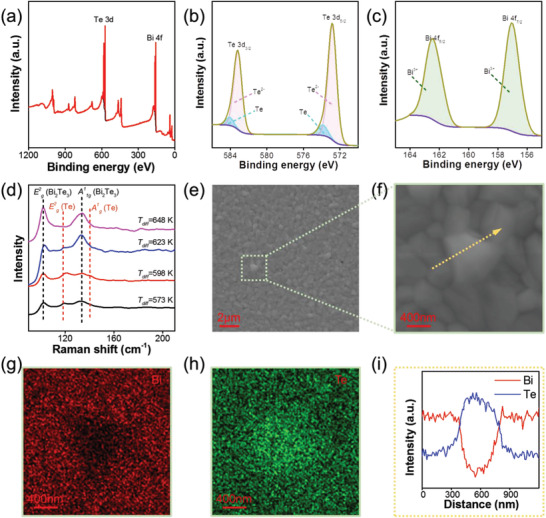
a) XPS full spectra survey, b) the Te 3d spectra, and c) the Bi 4f spectra of the Bi_2_Te_3_‐based thin film prepared at the diffusion temperature of 648 K. d) Raman spectra of the Bi_2_Te_3_ films prepared at the diffusion temperature of 573, 598, 623, and 648 K, respectively. e) High magnification and f) low magnification SEM images of the Bi_2_Te_3_ film prepared at the diffusion temperature of 623 K with corresponding EDS maps of g) Bi and h) Te, and i) EDS line scan spectra.

To further characterize the formation of Te‐embeddings, **Figure** **3**a presents the XPS spectrum of Bi_2_Te_3_ thin film prepared at the diffusion temperature of 623 K. The binding energy corresponding to Bi and Te are clearly detected. Figure 3b shows the observed peaks at 583.18 and 572.88 eV, which can be mainly ascribed to the binding energy of Te 3d_3/2_ and Te 3d_5/2_ deriving from Te^2−^ of Bi_2_Te_3_, respectively.^[^ ^36^ ^]^ The deviation between the detected Te spectrum and the Te^2−^ standard spectrum of Bi_2_Te_3_ is induced by Te^0^ (blue area), which proves the existence of Te. The binding energy detected at 162.48 and 157.08 eV should be attributed to the Bi 4f_5/2_ and Bi 4f_7/2_ (Figure 3c), respectively, which is consistent with that of Bi_2_Te_3_.^[^ ^39^ ^]^ Raman spectroscopy ranging from 90 to 210 cm^−1^ is also employed to study the composition changes of the Bi_2_Te_3_ thin films prepared at the diffusion temperature range of 573–648 K as shown in Figure 3d. The peaks located at 103.0 and 134.3 cm^−1^ are attributed to the E^2^_g_ and A^1^_1g_ modes, respectively, which are in agreement with the rhombohedra structure of Bi_2_Te_3_.^[^ ^40^ ^]^ The E^2^_g_ (121.6 cm^−1^) and A^1^_g_ (140.2 cm^−1^) peaks are assigned to the phonon mode of Te phase.^[^ ^40^ ^]^ With increasing diffusion temperature, the E^2^_g_ and A^1^_g_ peaks gradually disappeared at the diffusion temperature of 648 K indicating disappeared Te. Figure 3e–i shows the low‐ and high‐magnification SEM images with corresponding energy‐dispersive spectrometer (EDS) maps and line scan spectra of the Bi_2_Te_3_ film prepared at the diffusion temperature of 623 K. As can be seen, the observed Te precipitate in the size of ≈400 nm.

**Figure 4:**
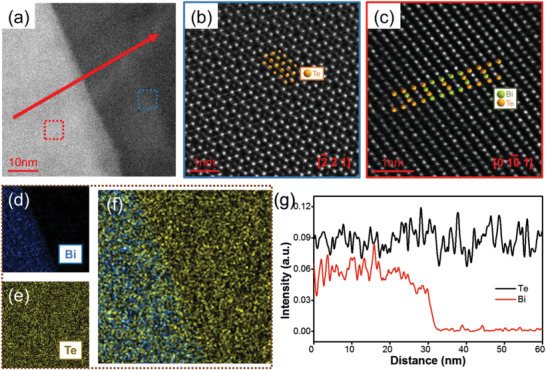
TEM analysis of Bi_2_Te_3_ thin films prepared at 623 K. a) High magnification TEM image. HRTEM of b) the blue dotted frame and c) the red dotted frame. d) EDS maps for d) Bi, e) Te, and f) total elements of the Bi_2_Te_3_ films taken from a). g) EDS scan lines for Bi and Te of the Bi_2_Te_3_ films along the red line taken from a).

To further characterize the crystal structure of as‐prepared Bi_2_Te_3_ thin films, TEM image of a typical sample prepared under the diffusion temperature of 623 K is shown in **Figure** **4**a, which clearly shows two typical grains of Te and Bi_2_Te_3_, respectively. Figure 4b shows the high‐resolution TEM (HRTEM) image of the right part of Figure 4a, which can well‐superimpose with the crystal structure of Te along [$\bar{2}$21] zone axis. Corresponding HRTEM image of the left part of Figure 4a is presented in Figure 4c and can well‐superimpose with the crystal structure of Bi_2_Te_3_ along [01$\bar{0}$1]. This indicates the left and right part of Figure 4a are highly crystallized Te and Bi_2_Te_3_, respectively. Figure 4d–f shows corresponding EDS maps. Te can be observed across the whole area. Bi can only be characterized on the left part. EDS line scan further clarified this point as shown in Figure 4g. This suggests the left part of Figure 4a is composed of both Bi and Te and should be Bi_2_Te_3_, and the right part of Figure 4a is composed of only Te (should be Te), which are consistent with the HRTEM analyses. In addition, Te‐rich Bi_2_Te_3_ with dense precipitates can make the grain boundaries and interfaces dense, introduce additional dislocations and point defects, and correspondingly strengthen phonon scattering leading to improved thermoelectric performance.^[^ ^20^ ^]^

**Figure 5:**
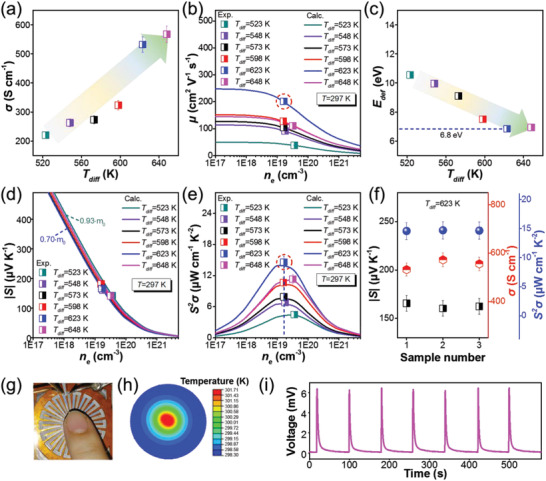
a) Measured *σ* of as‐prepared Bi_2_Te_3_‐based flexible thin films as a function of diffusion temperature. b) Measured *μ* as a function of *n*_e_ in comparison with the SPB model calculated values. c) *E*_def_ as a function of diffusion temperature. d) Measured |*S*| as a function of *n*_e_ in comparison with the SPB model calculated values. e) Measured *S* ^2^ *σ* as a function of *n*_e_ in comparison with the SPB model calculated values. f) Repeatedly measured room‐temperature *σ*, |*S*| and *S* ^2^ *σ* of Bi_2_Te_3_ flexible thin film prepared under the diffusion temperature of 623 K. g) Optical photo of a finger contact on thin film thermoelectric sensor with corresponding h) calculated temperature distribution and i) the output voltage signals.

Room‐temperature thermoelectric performance of as‐prepared Bi_2_Te_3_ thin films is shown in **Figure** **5**. Figure 5a shows the *σ* of the Bi_2_Te_3_ thin films as a function of diffusion temperature, which increases with increasing the diffusion temperature. The *σ* value increases with increasing the diffusion temperature, which approaches as high as 567.69 S cm^−1^ at the diffusion temperature of 648 K. To understand the evolution of *σ* with increasing diffusion temperature, Figure 5b presents the *n*_e_‐dependent *μ* of as‐prepared Bi_2_Te_3_‐based thin films in comparison with the single parabolic band (SPB) model calculated values. The calculation process of the SPB model is shown in Equations (S1)–(S5) of the Supporting Information. As can be seen, the *μ* dramatically increases while the *n*_e_ stays around 2–4 × 10^19^ cm^−3^, which indicates the increasing *μ* is not induced by the reduced *n*_e_. The *μ* approaches the maximum of ≈200 cm^2^ V^−1^ s^−1^ at the diffusion temperature of ≈623 K. Figure 5c displays the calculated deformation potential coefficient (*E*_def_) as a function of diffusion temperature. The *E*_def_ decreases with increasing temperature and also minimizes at the diffusion temperature of ≈623 K, which is consistent with the maximized *μ* at the same diffusion temperature. This should be attributed to the Bi_2_Te_3_/Te heterostructure, which induced energy filtering effect and correspondingly increased carrier energy and *µ*.^[^ ^41^, ^42^ ^]^ Figure 5d shows the *n*_e_‐dependent |*S*| of as‐prepared n‐type Bi_2_Te_3_‐based thin films in comparison with the SPB model calculated values. With increasing diffusion temperature, the **|** *S* **|** increased from ≈140 µV K^−1^ at the diffusion temperature of 523 K to ≈165 µV K^−1^ at the diffusion temperature of 623 K. The high |S| at 623 K is attributed to the strong energy filtering effect at the Bi_2_Te_3_/Te interfaces with the maximum element Te content. Further increasing the diffusion temperature to 648 K oppositely reduces the |*S*| due to sufficiently reacted Te. The minor change of *S* with the diffusion temperature lower than 623 K can be mainly attributed to the change of *n*_e_ regardless of the slightly varying effective mass (m^*^) ranging from 0.7 to 0.93 m_0_. Figure 5e shows the *n*_e_‐dependent *S* ^2^ *σ* of as‐prepared n‐type Bi_2_Te_3_‐based thin films in comparison with the SPB model calculated values, where the *S* ^2^ *σ* approaches as high as 14.58 µW cm^−1^ K^−2^ at the diffusion temperature of 623 K. This should be mainly ascribed to the energy filtering effect boosted *μ* while the *n*_e_ is close to the optimized level. Figure 5f presents the repeatedly measured *σ*, |*S*|, and *S* ^2^ *σ* of Bi_2_Te_3_ flexible thin film prepared under the diffusion temperature of 623 K. As can be seen, thermoelectric performance of as‐prepared Bi_2_Te_3_ flexible thin film remains closely consistent during the repeating measurement process, which indicates high stability of the film. The evaluation of repeatability of *σ*, *S*, and *S* ^2^ *σ* of Bi_2_Te_3_ flexible thin film is further shown in Figure S3 (Supporting Information) also indicating high repeatability and stability in their thermoelectric performance. To demonstrate the applicability of our n‐type Te‐embedded Bi_2_Te_3_ flexible thin films, we have assembled a demonstrating sensor with corresponding p‐type Sb_2_Te_3_ legs (thermoelectric performance is shown in Table S2, Supporting Information) as shown in Figure 5g. Touching the electrode with finger can induce a temperature difference between the center and the surrounding area as simulated in Figure 5h and correspondingly induce electrical signals. Continuous response of the thermoelectric sensor is measured for 7 cycles. The time interval between peak and valley of each pulse is ≈1 s, which includes device response time and finger heat transfer time. The output signals of the thermoelectric sensor show a fast on‐off response as shown in Figure 5i, where the state of finger touching is “on,” and the state of finger moving away is “off.”

## 3 Conclusion

In this study, we have successfully prepared n‐type Bi_2_Te_3_ flexible thin films on flexible PI substrates by a novel thermal diffusion reaction method. Reaction control can lead to the formation of Te‐embeddings and Te/Bi_2_Te_3_ heterostructures in the Bi_2_Te_3_ thin films, which theoretically can induce energy filtering effect due to the Δ*E_g_* between Bi_2_Te_3_ and Te valence bands. Experimentally, the reaction condition and formation of Te embeddings can be engineered through adjusting the diffusion temperature. An excellent *S* ^2^ *σ* of 14.58 µW cm^−1^ K^−2^ has been achieved at room temperature due to Te‐embeddings induced energy filtering effect and corresponding ultrahigh *μ* of ≈200 cm^2^ V^−1^ s^−1^. Our prepared n‐type Te‐embedded Bi_2_Te_3_ flexible thin film possesses comparable thermoelectric performance comparing with state‐of‐art Bi_2_Te_3_‐based flexible thin films. This result well proves that tunning the diffusion temperature during the thermal diffusion process can effectively introduce embeddings and Te/Bi_2_Te_3_‐heterostructures and boost the thermoelectric performance of B_2_Te_3_ flexible thin films. The assembled thermoelectric sensor with fast response characteristics further proved the applicability of as‐prepared Te‐embedded Bi_2_Te_3_ thin films prepared by our thermal diffusion method.

## 4 Experimental Section

### Bi_2_Te_3_ Thin Film Preparation

The n‐type Bi_2_Te_3_ thin films were deposited on a flexible PI substrate using a thermal diffusion reaction method. First, Bi and Te thin films were deposited separately on PI substrates by using radiofrequency magnetron sputtering technology. The deposition parameters of the thin film are summarized in the Supporting Information. Then, as‐deposited Te and Bi thin films were tightly pressurized, under the external pressure of 5 × 10^6^ Pa in a vacuum (2 × 10^−3^ Pa) chamber followed by a heating process. The diffusion temperature was increased to the target temperature within 30 min and dwelled for 2 h, the samples were cooled down to room temperature under vacuum. The thickness of the thin films is ranging ≈550 nm and the measured results by using have been added in Table S1 (Supporting Information).

### Device Fabrication

First, Bi, Sb, and Te precursor films were deposited on the PI substrates. Subsequently, the flexible thermoelectric prototype sensor was fabricated using the thermal diffusion reaction method.

### Characterization of the Bi_2_Te_3_ Films

The crystal structures as‐prepared Bi_2_Te_3_ films were characterized by X‐ray diffraction (XRD, D/max 2500 Rigaku Corporation, CuK*α* radiation). The surface morphology and cross‐section microstructures were investigated by SEM (Zeiss supra 55). The compositions of Bi_2_Te_3_ films are analyzed by an EDS (Bruker Quantax 200). Samples for cross‐sectional TEM analysis were fabricated by a dual‐beam SEM‐FIB (focused ion beam, Scios, FEI). The crystal structures of as‐prepared Bi_2_Te_3_‐based flexible thin films were also investigated by the transmission electron microscope (TEM, FEI, Titan3 Themis G2). The chemical bonding was investigated by Raman spectra measurement system (Horiba Jobin Yvon). X‐ray photoelectron spectroscopy (XPS, Thermo Escalab 250XI) was used to study the valence states of the composing elements. Carrier concentration (*n*_e_) and mobility (*µ*) values were recorded from the Hall measurement system (HL5500PC, Nano metrics). The *σ* and *S* of Bi_2_Te_3_ film were simultaneously measured by the SBA458 (Nezsch). The error bars of the measured data were determined from the SBA458 (5% for *S*, 5% for *σ*, and 10% *S* ^2^ *σ*).

## Conflict of Interest

The authors declare no conflict of interest.

## Author Contributions

D.‐W.A., W.‐D.L., and Y.‐X.C. contributed equally to this work. D.‐W.A.: Investigation, Conceptualization, Methodology, Writing – original draft. W.‐D.L.: Investigation, Writing – review & editing. Y.‐X.C.: Writing – review & editing. M.W.: Investigation, Conceptualization, Methodology. B.J.: Investigation, Data curation, Validation. F.L.: Data curation, Validation. X.‐L.S.: Data curation, Validation. Z.‐H.Z.: Resources, Writing – review & editing, Supervision, Funding acquisition. G.L.: Resources, Writing – review & editing. X.‐H.Z.: Supervision. P.F.: Resources, Writing – review & editing, Supervision, Funding acquisition. Z.‐G.C.: Supervision, Writng – review & editing.

## Supporting information

Supporting Information

## Data Availability

Research data are not shared.
