# Supplementary material for: Novel Thermal Diffusion Temperature Engineering Leading to High Thermoelectric Performance in Bi2Te3‐Based Flexible Thin‐Films
Source: Adv Sci (Weinh). 2021 Dec 22;9(5):2103547. doi: 10.1002/advs.202103547 (PMC8844477; doi:10.1002/advs.202103547)
Supplement: Supplementary file 1 — Supporting Information [file ADVS-9-2103547-s001.pdf]

ADVANCED SCIENCE
Open Access

Supporting Information

for *Adv. Sci.,* DOI: 10.1002/advs.202103547

Novel thermal diffusion temperature engineering leading to high thermoelectric performance in Bi_2Te_3-based flexible thin-films

Dong-Wei Ao, Wei-Di Liu, Yue-Xing Chen, Meng Wei, Bushra Jabar, Fu Li, Xiao-Lei Shi, Zhuang-Hao Zheng, Guang-Xing Liang, Xiang-Hua Zhang, Ping Fan, Zhi-Gang Chen*

Novel thermal diffusion temperature engineering leading to high thermoelectric performance in Bi_2Te_3-based flexible thin-films

Dong-Wei Ao,[#] Wei-Di Liu,[#] Yue-Xing Chen,[#] Meng Wei, Bushra Jabar, Fu Li, Xiao-Lei Shi, Zhuang-Hao Zheng,[*] Guang-Xing Liang, Xiang-Hua Zhang, Ping Fan, Zhi-Gang Chen

Contents:

Fig. S1. (a) films prepared at 548 K and at 573 K. The photo Te represents the Te film after diffusion/reaction.

Fig. S2 Grain size corresponding to: (a) Fig. 2(d), (b) Fig. 2(e), (c) Fig. 2(f)

Figure S3. The evaluation of repeatability of (a) σ, (b) S, and (c) $S^2\sigma$ of n-type Bi_2Te_3 thin films prepared at 623 K.

Table S1 EDS and thickness results of Bi_2Te_3 thin films at various diffusion temperatures

Tab. S2 TE performance for *p*-type Sb_2Te_3 thin films prepared at 350 °C.

Experimental Details

The residual pressure was 7.0 × 10^{-4} Pa and the working pressure was 1.0 Pa with an argon flow of 40 Sccm during the thin film deposition. The sputtering parameters were as follows: the sputtering power of Bi (25 W) and Te films (27 W), the sputtering time of Bi (20 min) and Te films (27 min), and the sputtering rates of Bi (12.5 ± 1 nm/min) and Te films (13.3 ± 1 nm/min), respectively.

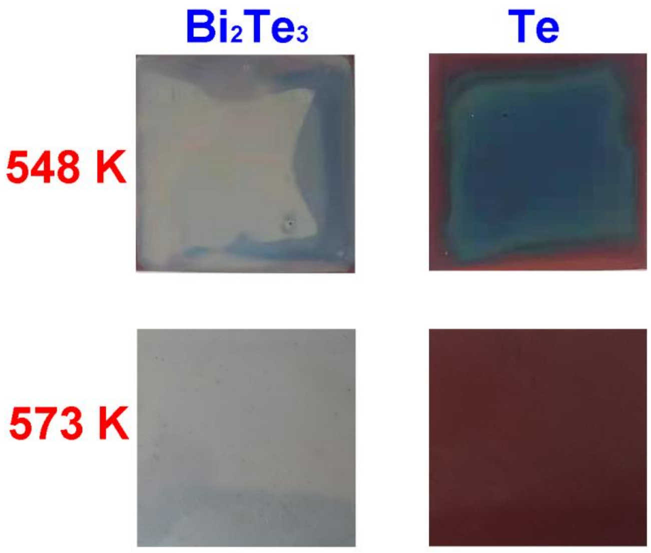

Fig. S1. (a) films prepared at 548 K and at 573 K. The photo Te represents the Te film after diffusion/reaction.

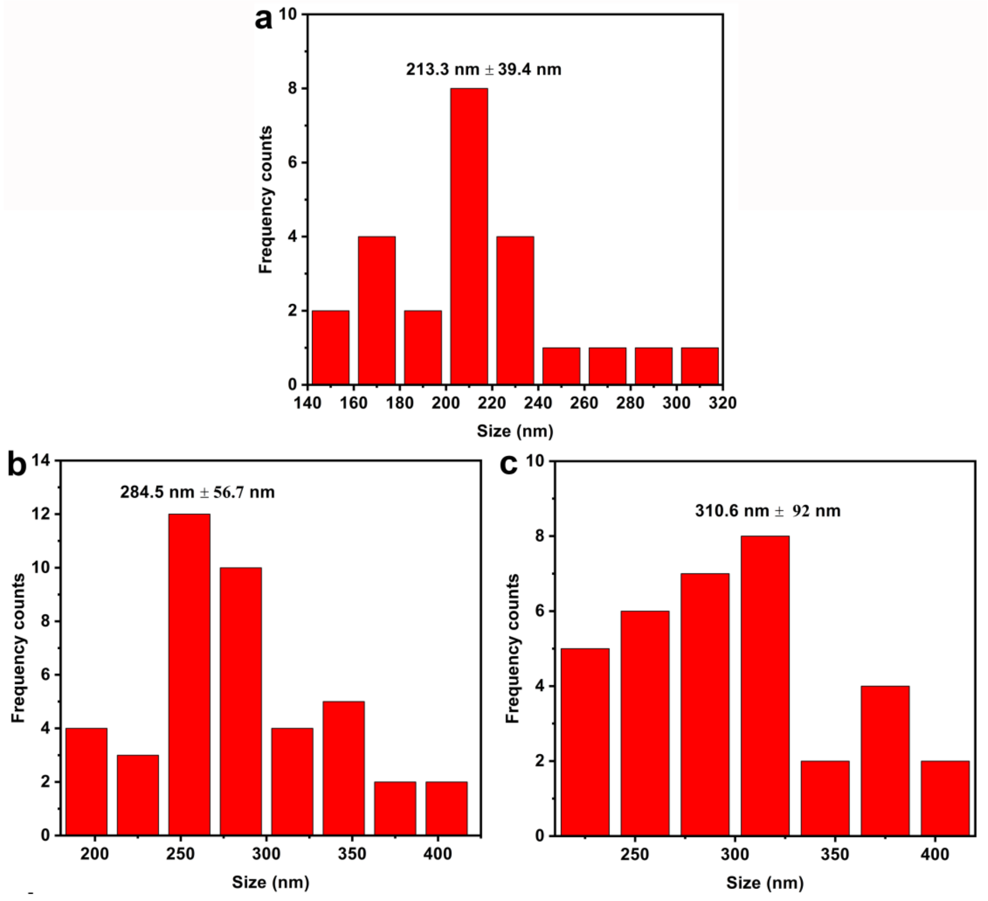

Fig. S2 Grain size corresponding to: (a) Fig. 2(d), (b) Fig. 2(e), (c) Fig. 2(f).

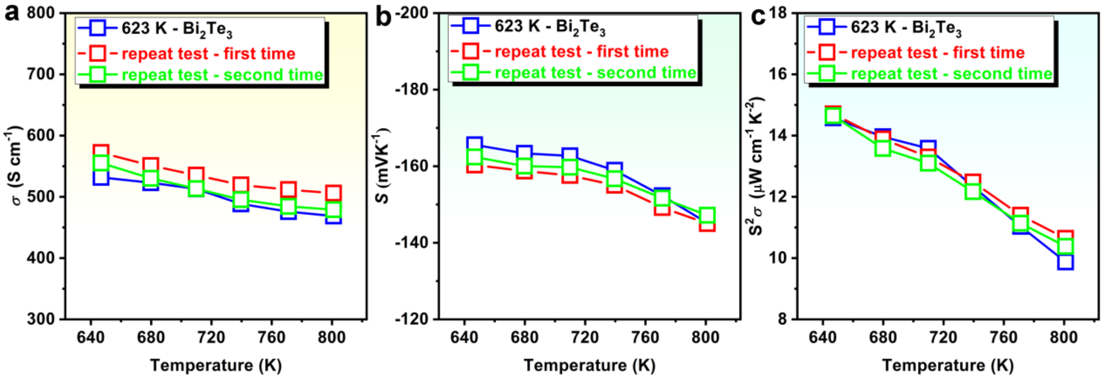

Figure S3. The evaluation of repeatability of (a) σ, (b) S, and (c) $S^2\sigma$ of n-type Bi_2Te_3 thin films prepared at 623 K.

Table S1 EDS and thickness results of Bi_2Te_3 thin films at various diffusion temperatures

Temperature (K)	523	548	573	598	623	648
Bi (At %)	44.97	33.70	36.40	35.67	38.09	39.14
Te (At %)	55.03	63.30	63.60	64.33	61.91	60.86
Bi : Te	2:2.45	2:3.45	2:3.49	2:3.61	2:3.25	2:3.11
Bi_2:Te_3+Te_x	x= -0.55	x=+0.45	x=+0.49	x=+0.61	x=+0.25	x=+0.11
Thickness (nm)	~525	~550	~580	~540	~570	~545

Table S2 TE performance for *p*-type Sb_2Te_3 thin films prepared at 623 K.

S (μVK^{-1})	σ (Scm^{-1})	PF (μW$cm^{-1}K^{-2}$)
115	1470	1.94

The calculation process of the SPB model is details:

The single parabolic band (SPB) model was used to simulate and analyze the transport properties as followed:

$$s(\eta) = \frac{k_B}{e} \cdot \left[\frac{\left(r + \frac{5}{2}\right) \cdot F_{r+\frac{3}{2}}(\eta)}{\left(r + \frac{3}{2}\right) \cdot F_{r+\frac{1}{2}}(\eta)} - \eta \right] \tag{S1}$$

$$n_H = \frac{1}{e \cdot R_H} = \frac{(2m^* \cdot k_B T)^{\frac{3}{2}}}{3\pi^2 \hbar^3} \cdot \left[\frac{\left(r + \frac{3}{2}\right)^2 \cdot F_{r+\frac{1}{2}}{}^2(\eta)}{\left(2r + \frac{3}{2}\right) \cdot F_{r+\frac{1}{2}}(\eta)} \right] \tag{S2}$$

$$\mu_H = \left[\frac{e\pi\hbar^4}{\sqrt{2}(k_B T)^{\frac{3}{2}}} \frac{C_1}{E_{def}{}^2 (m^*)^{\frac{5}{2}}} \right] \cdot \left[\frac{\left(r + \frac{3}{2}\right) \cdot F_{2r+\frac{1}{2}}(\eta)}{\left(r + \frac{3}{2}\right)^2 \cdot F_{r+\frac{1}{2}}(\eta)} \right] \tag{S3}$$

$$L=\left(\frac{k_{\mathrm{B}}}{e}\right)^{2}\left\{\frac{\left(r+\frac{7}{2}\right)\cdot F_{r+\frac{5}{2}}(\eta)}{\left(r+\frac{3}{2}\right)\cdot F_{r+\frac{1}{2}}(\eta)}-\left[\frac{\left(r+\frac{5}{2}\right)\cdot F_{r+\frac{3}{2}}(\eta)}{\left(r+\frac{3}{2}\right)\cdot F_{r+\frac{1}{2}}(\eta)}\right]^{2}\right\} \quad (S4)$$

$$F_i(\eta)=\int_0^{\infty}\frac{x^i}{1+e^{(\mathrm{x}-\eta)}}\mathrm{d}x \quad (S5)$$

where η is the reduced Fermi level, k_B is the Boltzmann constant, e is the electron charge, r is the carrier scattering factor (r = $-1/2$ for acoustic phonon scattering) [2], R_{H} is the Hall coefficient, m^* is the effective mass, $\hbar$ is the reduced Plank constant, C_1 is the elastic constant for longitudinal vibrations, E_{def} is the deformation potential coefficient, $F_i(\eta)$ is the Fermi integral. And $C_1 = v_1^2\rho$, where v_1 = 2600 m s^{-1} is the longitudinal sound velocity [3]."

To clarify the calculation of deformation potential, we have added the following discussion on Page 18 of the revised manuscript: "The calculation process of the SPB model is details in Equation S1-S5". Correspondingly, the calculation details have added in the supporting information.

Reference

[1] W. D. Liu, X. L. Shi, M. Hong, L. Yang, R. Moshwan, Z. G. Chen, J. Zhou. *J. Mater. Chem. C.* **2018**, 6,13225

[2] O. Ivanov, M. Yaprintsev, *Solid State Sci.* **2018**, 76, 111-117.

[3] Y. G. Wang, C. Liebig, Xianfan Xu, R. Venkatasubramanian, *Appl. Phys. Lett.* **2010**, 97, 083103.
